# Primary succession of Bifidobacteria drives pathogen resistance in neonatal microbiota assembly

**DOI:** 10.1038/s41564-024-01804-9

**Published:** 2024-09-06

**Authors:** Yan Shao, Cristina Garcia-Mauriño, Simon Clare, Nicholas J. R. Dawson, Andre Mu, Anne Adoum, Katherine Harcourt, Junyan Liu, Hilary P. Browne, Mark D. Stares, Alison Rodger, Peter Brocklehurst, Nigel Field, Trevor D. Lawley

**Affiliations:** 1 Host–Microbiota Interactions Laboratory, Wellcome Sanger Institute, Hinxton, UK; 2 Institute for Global Health, University College London, London, UK; 3 Birmingham Clinical Trials Unit, University of Birmingham, Birmingham, UK

**Keywords:** Microbiome, Metagenomics, Microbial ecology

## Abstract

Human microbiota assembly commences at birth, seeded by both maternal and environmental microorganisms. Ecological theory postulates that primary colonizers dictate microbial community assembly outcomes, yet such microbial priority effects in the human gut remain underexplored. Here using longitudinal faecal metagenomics, we characterized neonatal microbiota assembly for a cohort of 1,288 neonates from the UK. We show that the pioneering neonatal gut microbiota can be stratified into one of three distinct community states, each dominated by a single microbial species and influenced by clinical and host factors, such as maternal age, ethnicity and parity. A community state dominated by *Enterococcus faecalis* displayed stochastic microbiota assembly with persistent high pathogen loads into infancy. In contrast, community states dominated by *Bifidobacterium*, specifically *B. longum* and particularly *B. breve*, exhibited a stable assembly trajectory and long-term pathogen colonization resistance, probably due to strain-specific functional adaptions to a breast milk-rich neonatal diet. Consistent with our human cohort observation, *B. breve* demonstrated priority effects and conferred pathogen colonization resistance in a germ-free mouse model. Our findings solidify the crucial role of Bifidobacteria as primary colonizers in shaping the microbiota assembly and functions in early life.

## Abstract (web summary)

Primary colonization by microbial communities dominated by Bifidobacteria contribute to stable gut microbiota assembly and long-term pathogen resistance in neonates.

## Main

Human gut microbiota colonization commences immediately at birth when neonates are exposed to microorganisms from the surrounding environment and maternal sources (for example, gut^1–5^, vagina^2–4^, skin^3,4^, breast milk^3,6^). We recently reported in the UK Baby Biome Study (BBS) that maternal transmission of primary colonizers, such as commensal *Bifidobacterium* and *Bacteroides* species, is disrupted in caesarean-section (CS) and antibiotic-exposed births, instead predisposing the neonatal gut microbiota (NGM) to colonization by antibiotic resistant healthcare-associated pathogens^1^. This observation suggests the possibility of ‘priority effects’ in human gut microbiota assembly, which posits the arrival order of primary colonizer species determines the outcome of the microbiota assembly during a primary ecological succession (from sterile to complex communities)^7,8^. The NGM represents the earliest window of opportunity for intervention with probiotics or prebiotics to prevent or restore impaired microbiota development. However, little is known about the ecological priority effects in the NGM assembly due to a lack of high-resolution, longitudinal human microbiome data from the neonatal period (that is, the first month of life).

## Results

**Extended Data Fig. 1:**
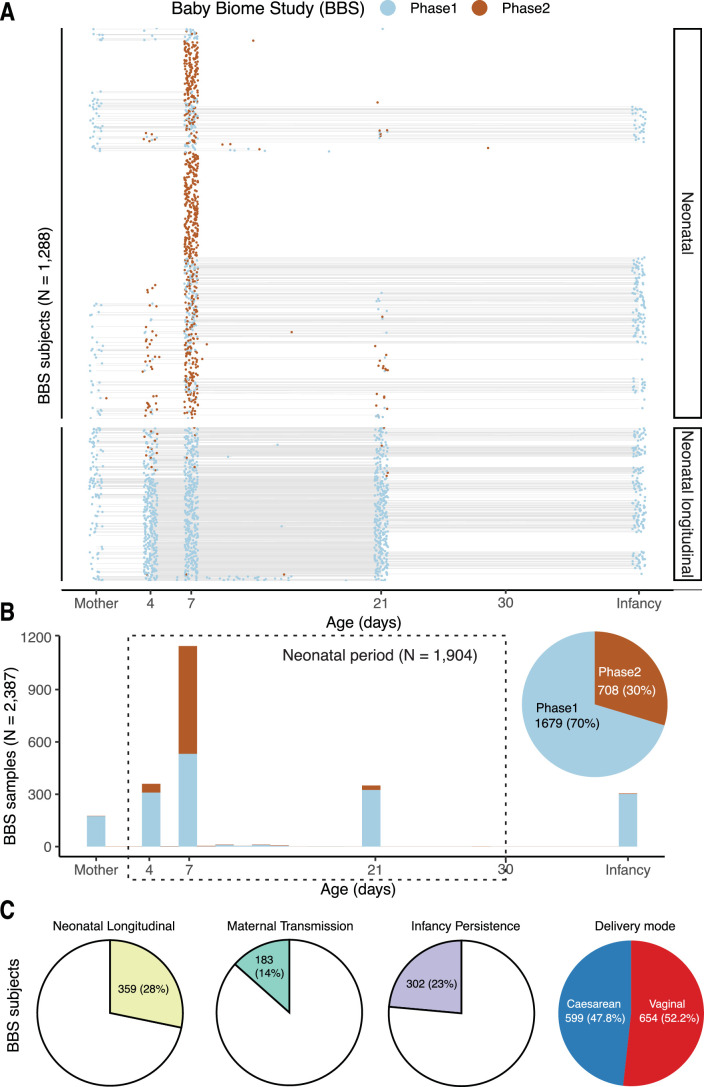
Overview of sampling in the Baby Biome Study. **(a-b)** Shotgun metagenomes of 2,387 faecal samples from 1,288 neonatal subjects across Phase 1 (Shao et al.^1^) and Phase 2 (this paper). The majority of samples (80% or 1,904) are from the neonatal period **(b)**, primarily taken on day 4 (N=360), day 7 (N=1,149), and day 21 (N=350). **(a)** Rows represent subjects with paired maternal samples (for ‘maternal transmission’ analysis), longitudinal samples taken during the neonatal period (for ‘neonatal longitudinal’ analysis), and samples from the infancy period (for ‘infancy persistence analysis’). These relationships are indicated by lines linking the samples, with summarised proportions in **(c)**.

To comprehensively examine NGM assembly dynamics, we expanded on phase 1 of our BBS cohort (BBS1)^1,9^ with an additional 688 neonatal participants (primarily day 7) in phase 2 (BBS2), effectively doubling our sampling effort. A large-scale, longitudinal metagenomic characterization of the combined BBS dataset, comprising 2,387 gut microbiota samples from 1,288 healthy UK neonates (≤1 month), enabled us to study neonatal microbiota assembly with unparalleled scale and resolution (Extended Data Fig. 1a,b and Supplementary Tables 1–3). To investigate the origin and both short-term and long-term stability of the NGM primary colonizers, we utilized three subgroups from the expanded BBS2 cohort. These included (1) 183 neonate–mother pairs (representing 14% of participants), referred to as investigating ‘maternal transmission’; (2) 359 participants with longitudinal sampling within the neonatal period (median = 3 samples per participant on days 4, 7 and 21; representing 28% of participants), referred to as investigating ‘neonatal longitudinal colonization’; and (3) 302 participants with paired samples taken both in the neonatal period and later in infancy (at 8.75 ± 1.98 months; representing 23% of participants), referred to as investigating ‘infancy persistence’ (Extended Data Fig. 1c).

Complementing the increased sample size, we have also updated extensive, high-quality clinical and sociodemographic metadata harmonized from BBS clinical record forms and hospital electronic records (Methods), thereby facilitating robust statistical and epidemiological assessment of primary succession patterns. Most neonates in this cohort (84.5%, *N* = 836) were at least partially breastfed by their mothers, with 44.1% being exclusively breastfed (*N* = 436). A large majority of participants at the time of infancy sampling were still being breastfed (86.2%; *N* = 199), with very few fully weaned (0.87%; *N* = 2). Only 11.3% (*N* = 123) received postnatal antibiotics during the first week of life (Supplementary Table 4).

### Three community states in the neonatal gut microbiota

**Fig. 1:**
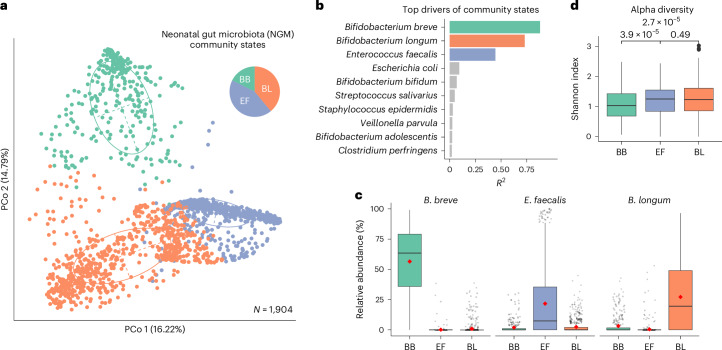
Dominant species driving three NGM community states. **a**, Principal coordinates analysis (PCoA) plots of 1,904 neonatal gut metagenomes sampled within the first 30 days of life and clustered using the PAM algorithm on the basis of species-level JSD. Three distinct NGM community states (optimal number clusters *k* = 3) were identified via PAM clustering. The inset pie chart displays the proportion of the three NGM community states, each labelled according to its primary driver species, namely *B. breve* (BB, green; *N* = 336, 17.6% of the samples), *E. faecalis* (EF, purple; *N* = 827, 43.4% of the samples) and *B. longum* (BL, orange; *N* = 741, 38.9% of the samples). Ellipses encapsulate 67% of the samples within each respective cluster. **b**, Top 10 driver species contributing to variation observed in the ordination space, as ranked by effect size (‘envfit’ *R*^2^, false discovery rate (FDR)-corrected two-sided test, *P* < 0.05). **c**,**d**, Each NGM community state is dominated by a single driver species, as measured by the high relative abundance of the driver species (**c**) and the low alpha diversity (**d**) across the three NGM community states (FDR-corrected, two-sided Wilcoxon test). Boxplot centre line and red point indicate the median and mean, respectively; box limits indicate the upper and lower quartiles; and whiskers indicate 1.5× the interquartile range (BB *n* = 336, EF *n* = 827, BL *n* = 741).

**Extended Data Fig. 2:**
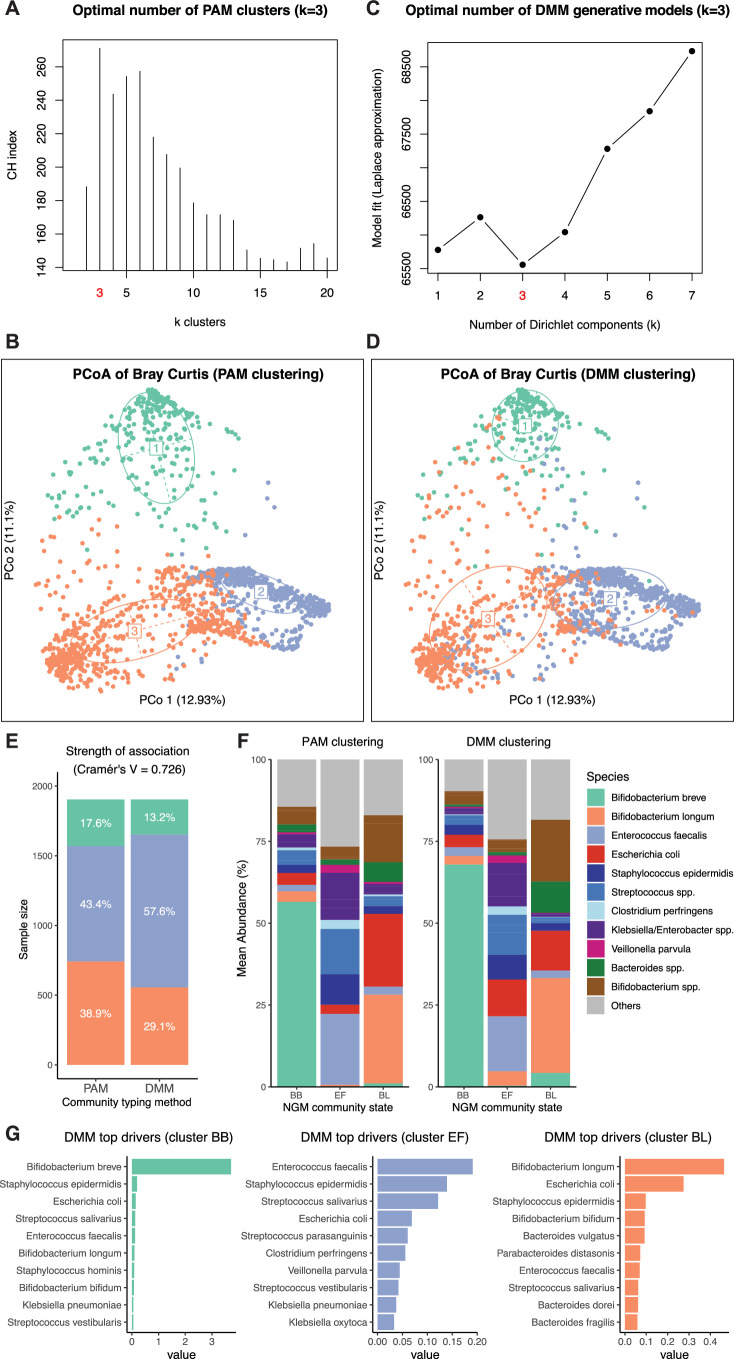
Consistency of NGM community state assignment across typing methods. **(a-d)** Identification of three NGM community states using both **(a)** Partitioning Around Medoids (PAM) clustering of JSD, with statistical support from the Calinski-Harabasz (CH) index, and **(c)** Dirichlet Multinomial Mixture (DMM) modelling using the Laplace approximation. (**b, d**) PCoA plots, representing 1,904 neonatal gut metagenomes, are color-coded by community state assignments, and based on species-level Bray-Curtis distances. **(e-g)** PAM-based and DMM-based community state assignment concordance: **(e**) Correlation between community state assignments shown with a Cramér's V correlation of 0.726. The proportions of community states assigned by each method are labelled. The breakdown of community states BB/EF/BL in PAM is 336/827/741, and in DMM is 252/1097/555. **(f)** Overlap in the dominant core species (≥1% mean abundance) in each community state, grouped at the genus level, with exceptions for the driver species *B. breve*, *E. faecalis* and *B. longum*. PAM-based assignment was chosen in downstream analyses given the higher relative abundances of these driver species in their respective community states (versus DMM-based assignment): *B. breve* 67.9% vs 56.5% (p<0.001), *E. faecalis* 21.7% vs 16.8% (p<0.001), and *B. longum* 27.25% vs 29.90% (p=0.24). Wilcoxon-test (two-sided) with FDR correction. (**g**) The top 10 driver species for each DMM-based community state are displayed, ranked by their assignment strength, as indicated on the y-axis.

**Extended Data Fig. 3:**
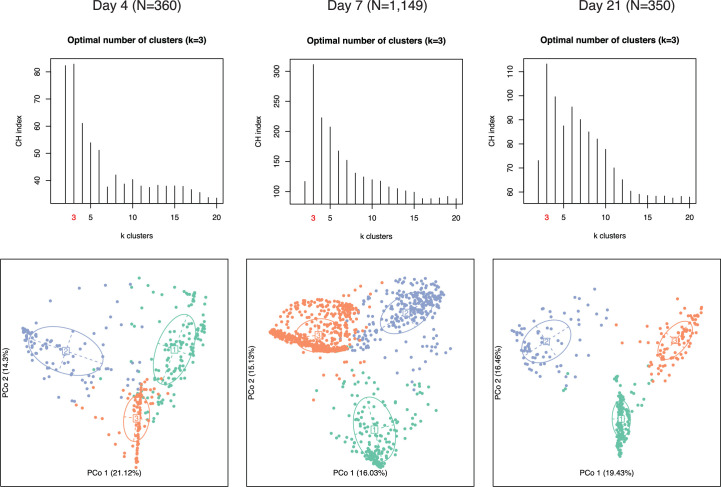
Consistency of NGM community state assignment across neonatal time points. Identification of three NGM community states using PAM-based clustering across three major time points in the neonatal period (day 4, N=360; day 7, N=1,149; day 21, N=350). PCoA plots, are color-coded by community state assignments and based on species-level JSD. Ellipses encapsulate 67% of the samples within each respective cluster.

To delineate the primary succession patterns of the NGM, we sought to identify the primary colonizers driving gut microbial community structure during the neonatal period. Applying partitioning around medoids (PAM) clustering to 1,904 BBS neonatal gut metagenomes at the species level revealed an optimal clustering of three within the NGM, hereafter referred to as ‘NGM community states’^10^ (Fig. 1a and Extended Data Fig. 2a,b). These three community states were further validated by another widely used microbial community typing method: the Dirichlet multinomial mixture (DMM) modelling framework (Extended Data Fig. 2c,d). Both the PAM and DMM-based approaches showed strong concordance in community state assignments (Cramér’s V correlation of 0.726; Extended Data Fig. 2e) and core species compositions (Extended Data Fig. 2f). Notably, these three community states were consistently observed across the three main sampling points in the BBS cohort (days 4, 7 and 21), underscoring their representativeness of the neonatal period, irrespective of the timing of sample collection (Extended Data Fig. 3).

**Extended Data Fig. 4:**
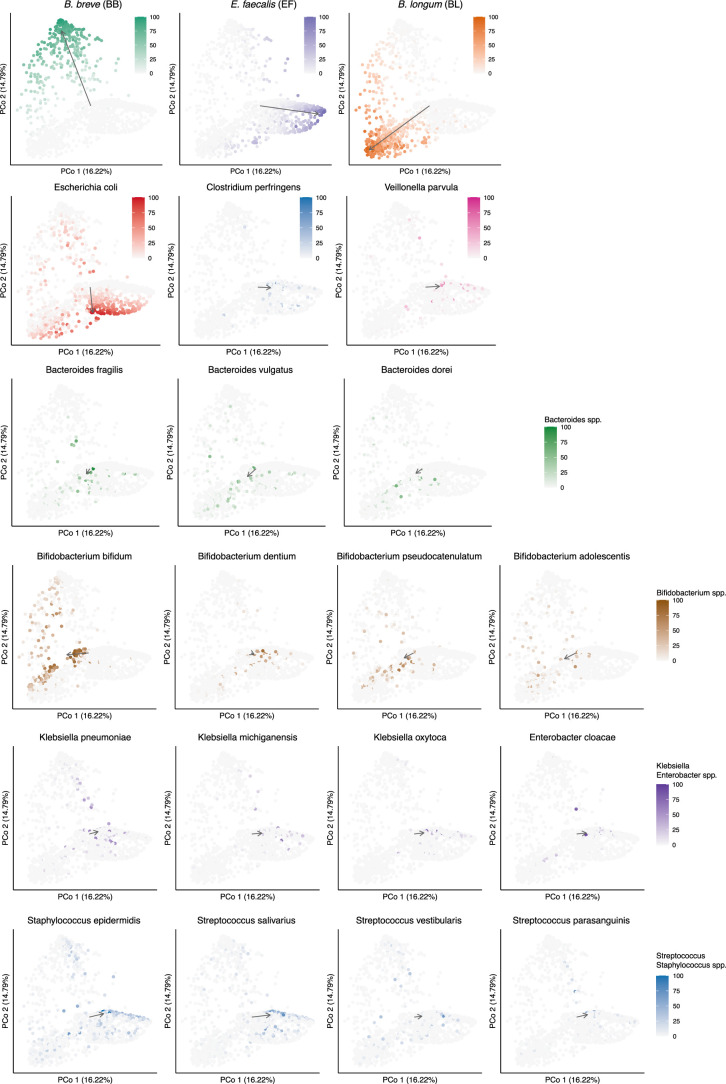
Abundance and co-occurrence of the NGM community state driver species. PCoA plots depicted in Fig. 1, with arrows, illustrate the scale and direction of core NGM species (>1% mean abundance) driving the formation of NGM community states (clusters). The length of the arrows is scaled to reflect the degree of contribution to the variation in NGM composition, with the arrow points towards increasing species abundance. Species that frequently co-occur with the NGM driver species within their respective community states share the same arrow direction.

Three bacterial species, *Bifidobacterium longum* subsp. *longum* (BL), *Bifidobacterium breve* (BB) and *Enterococcus faecalis* (EF) acted as the taxonomic drivers for each community state (Fig. 1b and Extended Data Figs. 2g and 4). Each species dominated their respective NGM community states with a relative mean abundance of 56.5% for BB, 21.7% for EF and 27.2% for BL (Fig. 1c). Henceforth, they are referred to as NGM driver species with acronyms indicating each respective community state.

**Extended Data Fig. 5:**
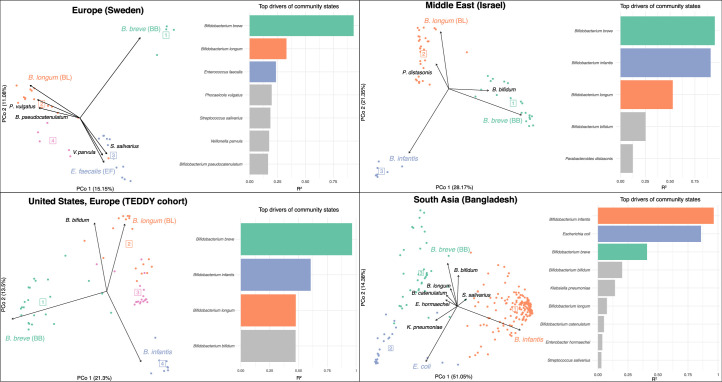
Validation of NGM community states and driver species across geographies and lifestyles. All three NGM community states and the driver species (*B. breve, B. longum or B. infantis, and E. faecalis*) were independently detected in infant gut metagenomic cohorts (0–6 months) from diverse geographical regions and lifestyles. These include Europe (Sweden, days 4–12, N=37), the United States (TEDDY cohort, months 2–6, N=69), the Middle East (Israel, weeks 1–24, N=60), and South Asia (Bangladesh, months 0–2, N=234). In the Bangladeshi cohort, which is a non-industrialised and non-urban population, the *B. infantis* and *E. coli*-driven clusters are representative of the *B. longum* (closely related to *B. infantis*) and *E. faecalis* (also facultative anaerobe opportunistic pathogen) community states, respectively. The analysis and visualization methods are consistent with those described in Fig. 1a, b.

The observed single-species dominance of either *B. breve*, *B. longum* or *E. faecalis* in very early life can also be consistently observed in other cohorts, albeit underreported owing to the previous undersampling during the neonatal period (the largest sample size being <100). Evidence for this comes from diverse populations and methodologies, including 16S gene or quantitative PCR (qPCR)-based observations in Norway^11^ (*N* = 87) and Denmark^12^ (*N* = 16), as well as shotgun metagenomic surveys of neonates across industrialized urban populations similar to the UK BBS cohort in Europe (Sweden^13^), Asia (Israel^14^) and North America (the TEDDY cohort^15,16^) (Extended Data Fig. 5). Importantly, the NGM community states observed across industrialized cohorts are paralleled in non-industrialized populations. In a peri-urban cohort in South Asia (Bangladesh^17^), although *B. breve* continues to be a primary NGM driver species, the community states typically driven by *B. longum* and *E. faecalis* in industrialized settings are instead represented by closely similar species: *B. infantis* (closely related to *B. longum*) and *Escherichia coli* (sharing facultative anaerobic and opportunistic pathogenic traits with *E. faecalis*). Collectively, these cross-study validations strengthen the generalizability of our results in neonatal populations from different geographical regions and lifestyles beyond the UK, and using different methodologies.

Of note, *B. longum* subsp. *infantis* (*B. infantis*), which is closely related to BL and often used as an infant probiotic, was not identified as a driver species. It was rarely detected (~2% prevalence based on 0.5% relative abundance) in the BBS neonates^14^. The near absence of *B. infantis* in our UK neonatal cohort aligns with findings from other Western industrialized countries, including a recent meta-analysis^14^ of cohorts from Israel, Sweden, Finland, Estonia, Italy and the USA^18^, where there is little evidence of *B. infantis* naturally colonizing the gut microbiota of healthy, full-term infants. This underscores the importance of distinguishing between closely related species that exhibit very different host colonization patterns.

**Extended Data Fig. 6:**
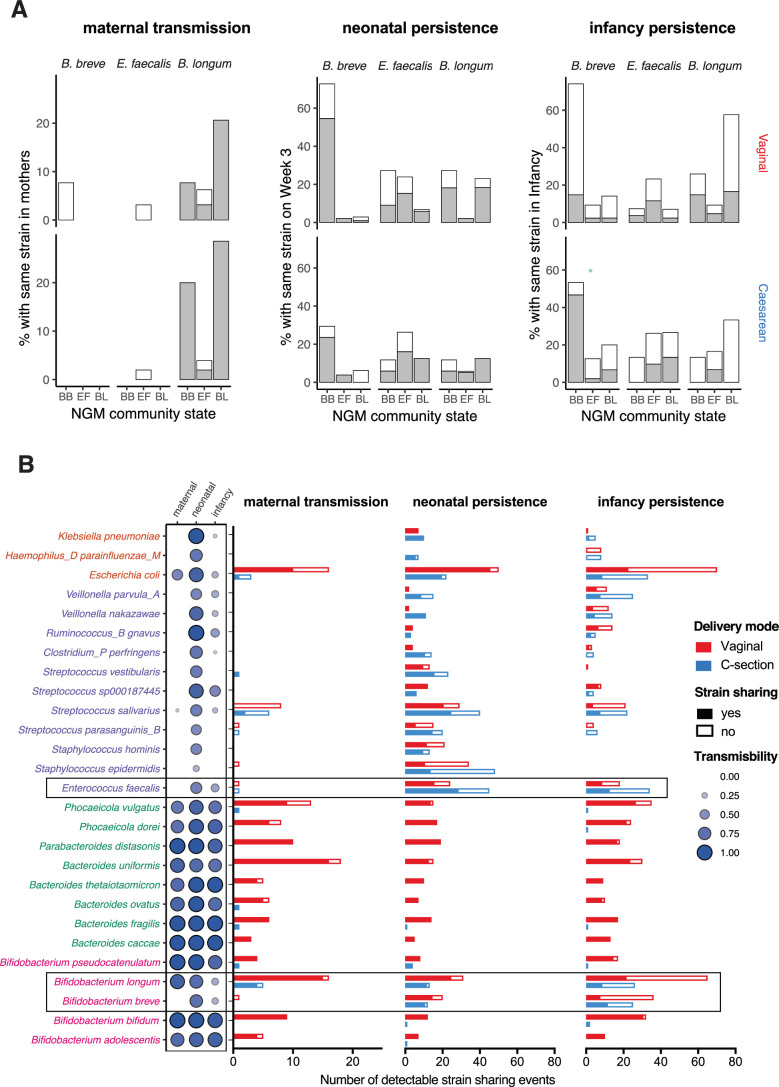
Strain-level dynamics and stability across NGM species. **(a)** Frequency of study participants detected with the same strains (in grey, otherwise in white) from their mother's faecal samples across NGM community states. To delineate transmission trends, the chart is categorized by birth mode and the three NGM driver species. Frequency of strain-sharing event (for example, maternal transmission in mother-baby pair or strain persistence within-individual longitudinal samples) is presented as raw counts of detectable strain sharing events normalized by the total number of subjects per birth mode and NGM community state (week 1). **(b)** Bar plots counting strain-sharing events across three settings: (Left) Maternal transmission in mother-infant dyads (183 subjects; 167 transmissions from 213 evaluated species-sample pairs). (Middle) Neonatal persistence via neonatal longitudinal sampling (359 subjects; 700 transmissions from 938 evaluated pairs). (Right) Infancy persistence from neonatal into infancy period (302 subjects; 464 transmissions from 920 evaluated pairs). When longitudinal samples were considered, strain sharing events were considered only once per subject per setting, using the time point with highest counts. Only species with ≥20 strain-sharing events detected across three settings are shown. Three community state driver species are highlighted in boxes. Transmission patterns often align with phylogeny: Actinomycetota/Actinobacteria (pink) and Bacteroidota/Bacteroidetes (green) typically transmit maternally during vaginal birth and persist into infancy. Conversely, Bacillota/Firmicutes (purple) and Pseudomonadota/Proteobacteria (orange) show lower maternal transmission rates and reduced neonatal persistence. Notable outliers include *E. coli* and *B. breve*. The size of bubbles represents the transmissibility of each species, which is its ratio of detected to potential strain-sharing events, as determined by StrainPhlAn4. Only subject pairs with sequencing depth sufficient for StrainPhlAn strain-level analyses are displayed; data points not shown are non-evaluable.

Applying metagenomic strain tracking analysis on the ‘maternal transmission’ subset, only *B. longum* exhibited evidence of maternal transmission, with all evaluable BL neonates (15 out of 15) harbouring the exact same *B. longum* strain found in their mothers’ gut microbiota. This result, consistent with a recent global meta-analysis^19^, strongly indicates the maternal gut microbiota as the main source of the BL community state (Extended Data Fig. 6). While we could have overlooked maternal transmission of very low-abundance *B. breve* and *E. faecalis* below the metagenomic strain detection limit, we consider it more likely that they originate from unsampled maternal (for example, *B. breve* in breast milk^20,21^) or environmental sources (for example, *E. faecalis* in the hospital birth environment^22,23^) previously implicated as potential sources of these species in the NGM.

The abundant dominance of single driver species was particularly pronounced in community state BB, in which *B. breve* constituted over half of the NGM by mean relative abundance, and exhibited the lowest microbial richness and evenness, as reflected by the alpha (Shannon) diversity (Fig. 1d). In comparison, the other two NGM community states, BL and EF, had higher microbial diversity, and other moderately abundant species frequently co-occurred with the driver species (Extended Data Fig. 2f,g); *B. longum* with commensal *E. coli*, *Bacteroides* and other *Bifidobacterium* species; *E. faecalis* with environment and skin-associated *Streptococcus*, *Staphylococcus* spp., as well as healthcare-associated opportunistic pathogens *Enterococcus*, *Klebsiella*, *Enterobacter* spp. and *C. perfringens*. Notably, these less-dominant species in EF were also known signatures of hospital CS birth not only in this UK cohort^1^ but also in cohorts from North America^24,25^, Latin America^24^ and Europe^13,24,26^.

### Factors influencing the acquisition of the NGM community states

**Fig. 2:**
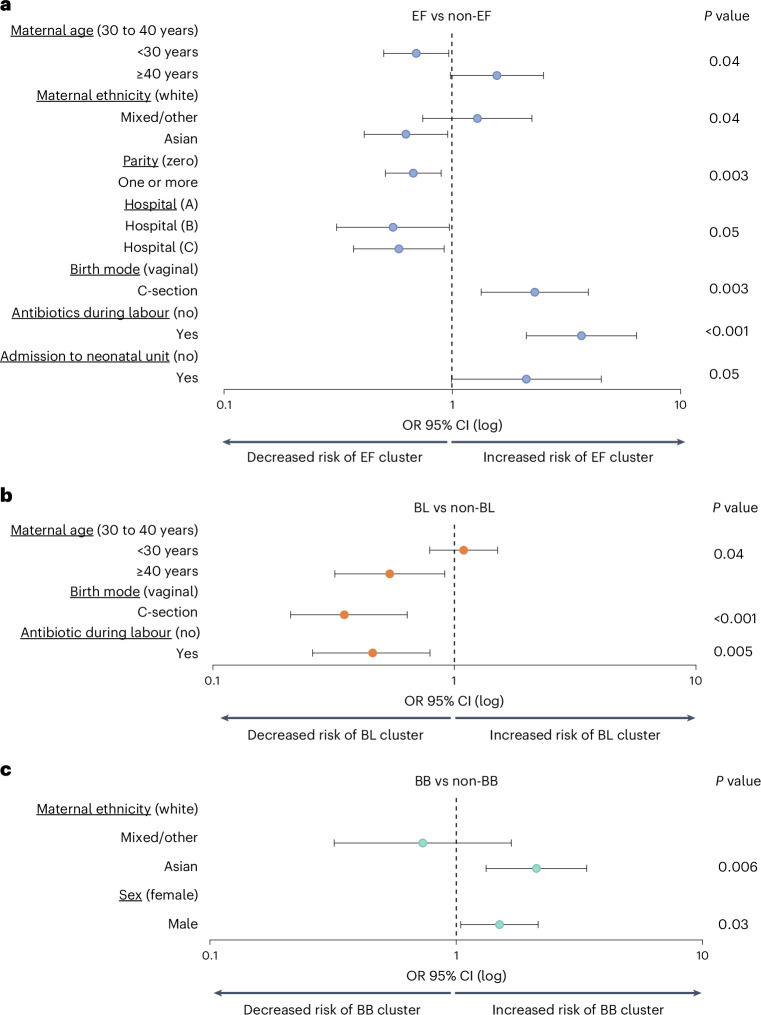
Clinical and sociodemographic variables associated with NGM community state in the first week of life (*N* = 1,108). **a**–**c**, Multivariate associations between clinical and sociodemographic variables and each week-1 NGM community state. Three different models were built: EF vs non-EF (**a**), BL vs non-BL (**b**) and BB vs non-BB (**c**). Likelihood ratio tests (two-sided) were used to calculate *P* values (without FDR correction), with *P* ≤ 0.05 in the multivariate models displayed. Odds ratios (OR) are plotted on a log_10_ scale. For details of univariate and multivariate analyses, refer to Supplementary Tables 5 and 6. The week-1 NGM community state was identified for each eligible participant using the earliest available sample from week 1, either on day 4 (*N* = 64) or day 7 (*N* = 1,044).

To determine the perinatal factors influencing the acquisition of each NGM community state, we performed epidemiological analyses using 20 high-quality clinical and sociodemographic metadata variables (*N* = 1,108 eligible participants; Fig. 2 and Supplementary Table 5). After adjusting for potential confounders in multivariate fixed-effect logistic regression models, we found that the acquisition of an EF community state was independently associated with being born via CS birth (compared to vaginal delivery (VD); adjusted odds ratio (AOR) = 2.30 [95% CI 1.34–3.95], *P* = 0.003; 70.5/23.6/40.0% among EF/BL/BB, respectively) and with the mother receiving intrapartum antibiotics during labour (AOR = 3.69 [95% CI 2.11–6.42], *P* < 0.001; 80.8/32.7/46.3% among EF/BL/BB, respectively). Conversely, being born via CS birth and labour antibiotics exposure were negatively associated with BL acquisition (AOR for CS vs VD = 0.36 [95% CI 0.21–0.64], *P* < 0.001; AOR for receiving antibiotics during labour = 0.46 [95% CI 0.26–0.79], *P* = 0.005, respectively).

Interestingly, several intrinsic host factors including sex (male with BB), maternal ethnicity (Asian with EF and BB), age (<30 and ≥40 with EF and BL, respectively) and parity (first time giving birth with EF) were also independently associated with specific community states. For example, mothers identifying as Asian (compared with white participants) were more likely to acquire BB (AOR = 2.11 [95% CI 1.32–3.38], *P* = 0.006) but less likely to acquire EF (AOR = 0.63 [95% CI 0.41–0.95], *P* = 0.04; 9.0/12.1/19.5% among EF/BL/BB, respectively). It is noteworthy that BB is the only community state that was exclusively influenced by host factors and independent of any clinical factors including mode of birth and antibiotics, which may suggest a distinct route of BB acquisition that remains unaffected by the perturbations associated with hospital births. These observations align with the hypotheses that maternal factors, such as genetic determinants of breast milk composition (for example, secretor status of the mothers)^27^, a history of previous pregnancies or cohabitation with children^28^, as well as cross-cultural differences in infant-care-associated behaviours^7^ may influence the vertical transmission of maternal microbiota.

Neither postnatal antibiotics nor breastfeeding exposure, whether immediately after birth or within the first week of life, appeared to predispose neonates to any specific community state. This lack of association is probably attributed to the uniformly high-levels of antibiotic-free status (84.7/90.8/89.5% among EF/BL/BB, respectively) and breastfeeding rates (79.1/81.8/88.6% among EF/BL/BB, respectively) during the earliest postnatal window sampled in this cohort. The absence of an association between breastfeeding and EF also aligns with previous reports that, despite its antimicrobial properties, breast milk alone does not inhibit *E. faecalis* growth in vitro^29,30^.

### Priority effects in NGM community state stability

**Fig. 3:**
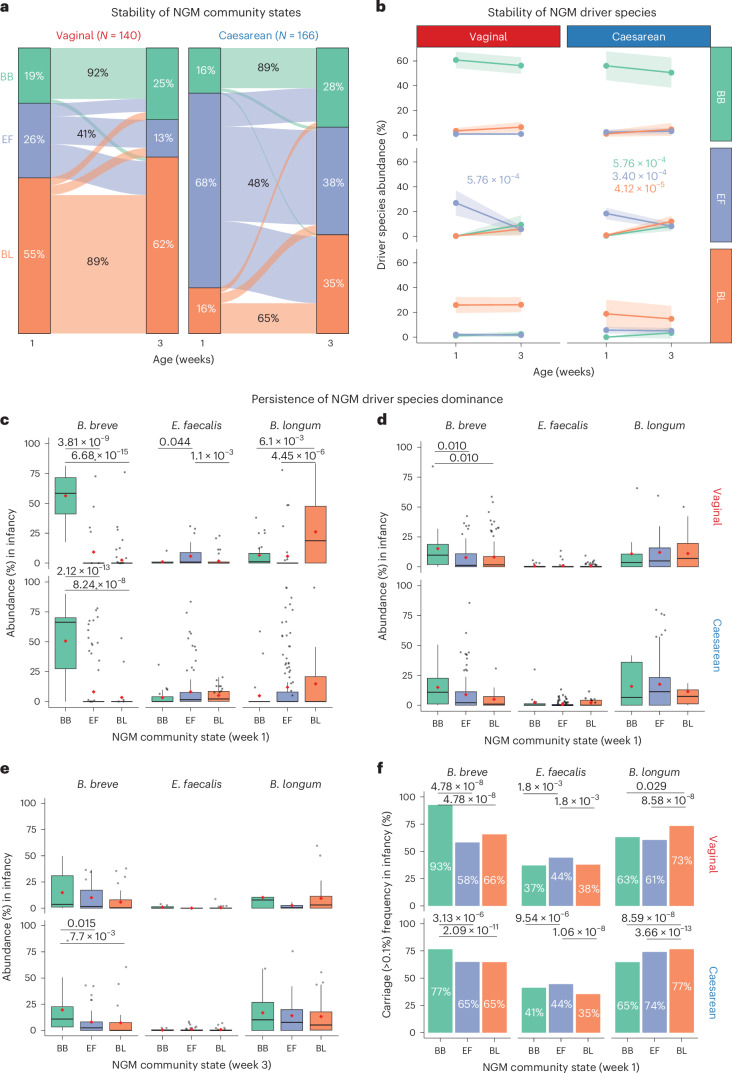
Dynamics and stability of NGM community states and their driver species. **a**,**b**, Stability of NGM community states (**a**) and levels of three species driving NGM community states (**b**) (week 1, based on the earlier sample of day 4 or 7) in neonates longitudinally sampled from weeks 1 to 3 (day 21, total *N* = 306; VD *N* = 140; CS *N* = 166). The proportion of community states that remained consistent from weeks 1 to 3 is depicted as a percentage of their initial sample size in week 1 (labelled in black). Participants starting with BB or BL on week 1 were significantly more likely to retain their community state in week 3 compared with those with EF (pairwise chi-square tests with FDR correction, *P* < 0.001). **c**–**e**, Persistence of the dominant abundance of driver species of NGM community states in week 1 (**c**,**d**) or week 3 (**e**) in the paired longitudinal samples obtained later at week 3 (**c**) and in infancy (**d**,**e**). **f**, Persistent carriage of week-1 driver species in paired longitudinal samples obtained later in infancy. Species carriage is defined using a threshold of 0.1% relative abundance. Sample sizes of participants longitudinally sampled for weeks 1 and 3 shown in **a**–**c** are: total *N* = 306; VD *N* = 26/39/75 among BB/EF/BL, respectively; CS *N* = 26/114/26 among BB/EF/BL, respectively; for week 1 and infancy (also referred to as the ‘infancy persistence’ group) shown in **d** and **f**: total *N* = 302; VD *N* = 27/43/90 among BB/EF/BL, respectively; CS *N* = 17/108/17 among BB/EF/BL, respectively; and for week 3 and infancy shown in **e**: total *N* = 146; VD *N* = 12/11/43 among BB/EF/BL, respectively; CS *N* = 17/37/26 among BB/EF/BL, respectively. Colour represents NGM community states or driver species: BB and *B. breve* in green; EF and *E. faecalis* in purple; BL and *B. longum* in orange. Boxplots as in Fig. 1. Statistical differences in abundance between time points (**a**), species (**c**–**e**) and carriage frequency (**f**) were determined using paired *t*-tests, Wilcoxon tests and chi-square tests (all two-sided) with FDR correction, respectively.

**Extended Data Fig. 7:**
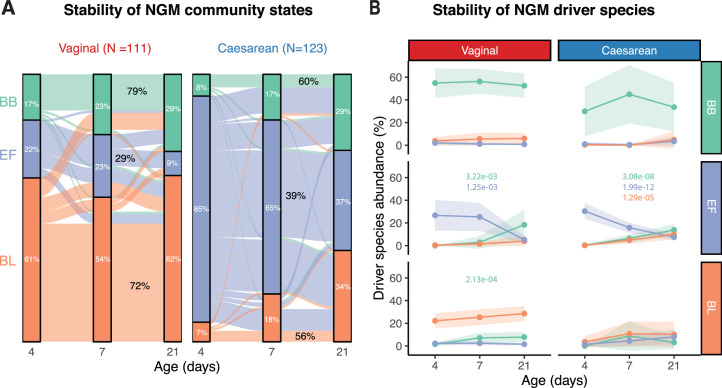
Colonisation dynamics in neonatal longitudinal samples. **(a)** Overview of NGM community states of all subjects individually sampled on major neonatal period sampling points day 4, 7 or 21, stratified by birth mode. In VD, N=176/602/156 on day 4, 7, and 21, respectively; In CS, N=184/547/194 on day 4, 7, and 21, respectively. Total samples N=1859. **(b)** Longitudinal shifts in NGM community states and the levels of driver species from week 1 to week 3, based on subjects longitudinally sampled across days 4, 7, and 21, N=234; VD, N=111; CS, N=123). Community states that remained consistent from first (day 4) to the final neonatal longitudinal sampling (day 7 or/and 21), is depicted as a percentage of their starting pool size (labelled in black). Subjects that began with either BB or BL community state on day 4 were significantly more likely to remain in the same community state on day 7 and 21, compared to those that began with EF (pairwise chi-squared tests with FDR correction, q-values < 0.01). However, this trend was not observed as early as day 7 (global chi-squared test, p=0.7043). The colour scheme represents the community states or driver species: BB and *B. breve* in green; EF and *E. faecalis* in purple; BL and *B. longum* in orange. Statistical differences in species abundance between longitudinal samples was determined using paired ANOVA test (two-sided) with FDR correction. Boxplot center line and red point indicate the median and mean, respectively; box limits indicate the upper and lower quartiles; and whiskers indicate 1.5× the interquartile range.

We reasoned that the three primary colonizers as NGM drivers could benefit from priority effects, which would be evident through the exclusion of, or replacement by, later-arriving species in the NGM. To search for evidence of such priority effects, we sought to examine the stability and temporal signals of both the NGM community states and their driver species in the ‘neonatal longitudinal’ subset, stratified by birth modes. Most VD neonates who initially acquired a *Bifidobacterium*-dominated community state (either 92% for BB or 89% for BL, 79% or 72% by considering transient switches between day 4 and 7) during week 1 retained their community state when resampled in week 3 (Fig. 3a and Extended Data Fig. 7a). By contrast, EF was the most unstable community state, with less than half of the neonates (29% in VD and 39% in CS) remaining in their early EF community state during the neonatal period (EF vs BB AOR 16.2 [95%CI 3.84–68.10], EF vs BL AOR 13.89 [4.02–48.02]; *P* < 0.001; Supplementary Table 6). Irrespective of birth mode, BB proved more stable than EF (pairwise chi-square test, corrected *P* < 0.001), while the sample size was insufficient to be confident about the relative stability of BL in CS neonates (65% versus 48% for EF; pairwise chi-square test, corrected *P* = 0.52).

The stability of the underlying driver species closely mirrored the observed community state dynamics. In contrast to *E. faecalis*, which rapidly declined throughout the stochastic assembly trajectory of the early community state EF, both *B. breve* and *B. longum* retained their high abundance within their respective community states throughout the 3-week neonatal sampling window (Fig. 3b and Extended Data Fig. 7b). Notably, both species, as late-arriving secondary colonizers (that is, colonized NGM only in week 3), exhibited signs of competitively excluding *E. faecalis* in CS neonates who initially acquired the EF community state (Fig. 3b). This competitive exclusion effect seemed most pronounced for *B. breve*; in contrast to *B. longum*, it was able to colonize VD neonates at increasing levels as a late-arriving species (Extended Data Fig. 7b). Among the primary colonizers that dominated the NGM in the first week, *B. breve* is the only species conferring durable colonization dominance (relative to the other driver species), which persisted as far as the final neonatal period sampling point at week 3 (*P* < 0.001 in VD and CS; Fig. 3c).

The stability of the two *Bifidobacterium* species is also reflected at the strain level (Extended Data Fig. 6); most of the neonates retained the same *B. longum* (79.5%, *N* = 35/44 BL neonates) or *B. breve* (75%, *N* = 24/32 BB neonates) strain they initially acquired throughout the neonatal period, in contrast to 62.3% for *E. faecalis* (*N* = 43/69 EF neonates; the denominators represent longitudinally sampled individuals with detectable strain sharing events).

Together, as primary colonizers, both *Bifidobacterium* species benefit from priority effects, maintaining a stable NGM assembly trajectory owing to their ability to confer durable species dominance and inhibit the later arrival of opportunistic pathogens such as *E. faecalis*. In particular, *B. breve* exhibits stronger priority effects between the two species (that is, only as a primary colonizer), as well as strong deterministic exclusion of *E. faecalis* (that is, as either a primary or a secondary colonizer).

### Stability of NGM driver species into infancy

We also assessed the longer-term engraftment of the NGM driver species in participants resampled 6–12 months beyond the neonatal period using the ‘infancy persistence’ subset. Remarkably, the relative dominance of *B. breve* (over the other driver species, in VD, *P* < 0.05; Fig. 3d) also extended into infancy when there was still no significant difference in breastfeeding rates between early NGM community states (BB/EF/BL: 88.4%/89.6%/80.5%, chi-square test, *P* = 0.18). In addition, the long-term competitive exclusion effect of *B. breve* was evident in CS neonates who either retained or transitioned into BB (primarily from EF) by week 3. These long-term stability patterns were exclusively observed for *B. breve*, with its abundance in infancy being almost double in neonates who previously had a BB community state compared with those with other community states (Fig. 3e).

Although NGM driver species rarely retained their differential abundance later in infancy (except *B. breve*), the frequency of carriage for all three driver species was consistently higher in infants stratified by their corresponding NGM community states (Fig. 3f). As many as 93% of VD (or 77% of CS) neonates with week-1 community state of BB still carried *B. breve*, compared with 58% and 66% (or 65% of CS) of VD neonates with week-1 community states EF and BL, respectively (pairwise chi-square tests, *P* < 0.001). While levels of *E. faecalis* in community state EF waned over time to non-differential levels later in infancy, neonatal acquisition of EF remains a predisposing factor for longer-term carriage of *E. faecalis*. This opportunistic pathogen species was still detected in higher proportions (44%) in neonates from the EF community state during their first week (relative to 37–41% in BB and 35–38% in BL) when resampled later in infancy, regardless of their birth mode (pairwise chi-square tests, *P* < 0.001; Fig. 3f).

### EF state enriched with virulence and antibiotic resistance genes

**Fig. 4:**
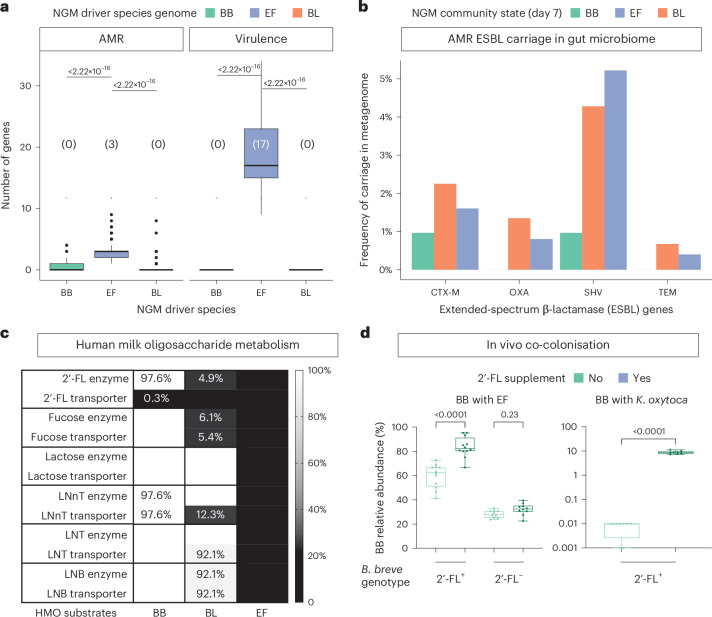
*Bifidobacterium* species drive resistance to antimicrobial resistance and pathogen colonization. **a**, Counts of detected AMR and virulence genes in driver species genomes, with median values enclosed in brackets. Wilcoxon test (two-sided) with FDR correction; number of genomes (isolates in brackets): BB *N* = 297 (30), EF *N* = 561 (54) and BL *N* = 391 (49). **b**, Carriage of high-risk AMR genes associated with ESBL in the day-7 NGM community state samples based on raw metagenomic assemblies (BB *N* = 207, EF *N* = 498, BL *N* = 444). The *x* axis shows the most clinically prevalent ESBL genes belonging to CTX-M, OXA, SHV and TEM families. **c**, Proportion of species genomes, indicated by a colour gradient, predicted to utilize HMOs or their primary downstream products, lactose and fucose. The actual proportions are labelled for genotypes that are not completely present. The predictions are based on the presence of both the gene and its encoded transporters required for utilization of each substrate. 2′-fucosyllactose (2′-FL) liberates lactose and fucose which are also present in breast milk. Utilizations of LNnT, LNT and LNB will all liberate lactose. **d**, NGM driver species BB confers pathogen colonization resistance in vivo. The boxplot depicts the relative abundance of BB compared to the opportunistic pathogen species EF or *K. oxytoca* (KO). The *x* axis represents three experimental groups co-colonized as follows: (1) BB type strain DSM 20213 (2′-FL^+^) with EF; (2) BB natural variant D19 (2′-FL^−^, isolated from a BBS neonate) with EF; and (3) BB type strain (2′-FL^+^) with KO (D63). The BB genotype (2′-FL^+/−^) indicates whether the strain encodes the α-l-fucosidase (GH95) enzyme encoding for 2′-FL metabolism. In each co-colonization group, one group of mice also received a 2′-FL supplement (50 mg ml^−1^ per day) in their daily drinking water. The *y* axis for BB co-colonization with KO is shown on a log scale. Each experimental condition included 3–5 mice per cage and 3 technical replicate cages. Statistical differences between treatment groups were determined using a *t*-test with Welch’s correction (two-sided). Boxplot centre line indicates the median, box limits indicate upper and lower quartiles, and whiskers indicate 1.5× the interquartile range.

To determine the functional differences among NGM community states, we leveraged their driver species as proxies for functional analyses, using 1,249 high-quality isolate (*N* = 133) and metagenome-assembled genomes (*N* = 1,116) generated from the corresponding community state samples (BB *N* = 297, EF *N* = 561, BL *N* = 391; Supplementary Table 7). We found a striking difference between *Bifidobacterium* spp. and *E. faecalis* functional profiles in antimicrobial resistance (AMR) and virulence potential. Importantly, all *E. faecalis* strain genomes recovered from neonates with EF community states encoded known virulence factors including 70% predicted to produce the toxin cytolysin^31^. By contrast, both *Bifidobacterium* driver species genomes displayed markedly reduced levels of AMR and virulence-associated genes, with a burden 10- to a 100-fold less than in EF (median 17 versus 0; Fig. 4a). Further AMR gene screening of the entire gut resistome within each community state revealed a higher carriage of high-risk AMR genes, such as CTX-M-15 linked to extended-spectrum beta-lactamase (ESBL), in both BL and EF community states (Fig. 4b). This underscores the notable pathogenic potential of ESBL-carrying Enterobacteriaceae pathogens co-occurring in non-BB community states. These findings align with our risk factor analyses (Fig. 2), which identified maternal antibiotics exposure during labour (to some VD and all CS neonates) as a strong risk for the acquisition of an EF community state that bears increased risk of AMR and virulence.

### Pathogen resistance of *B. breve* via metabolic adaptation to HMOs

**Extended Data Fig. 8:**
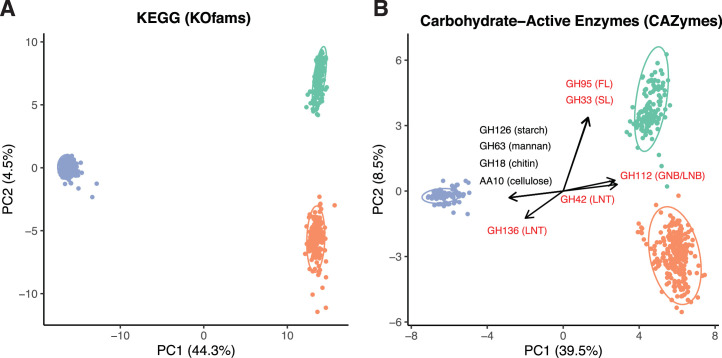
Species-driven functional divergence in NGM community states. **(a-b)** Principal Component Analysis (PCA) of community state driver enterotype species genomes. Groupings are based on the presence of genes tied to the full metabolic repertoire using **(a)** KEGG orthologs (KOfams) and carbon metabolism via **(b)** Carbohydrate-Active enZYmes (CAZymes). Each dot denotes an individual strain: *B. longum* (BL, N=342) in orange, *B. breve* (BB, N=267) in green, and *E. faecalis* (EF, N=507) in blue. Ellipses encapsulate the 95% confidence intervals. Arrows showcase the contribution of select CAZy genes to principal components (details in Extended Data Fig. 8). CAZy genes for human milk oligosaccharides (HMOs) utilisation are highlighted in red.

**Extended Data Fig. 9:**
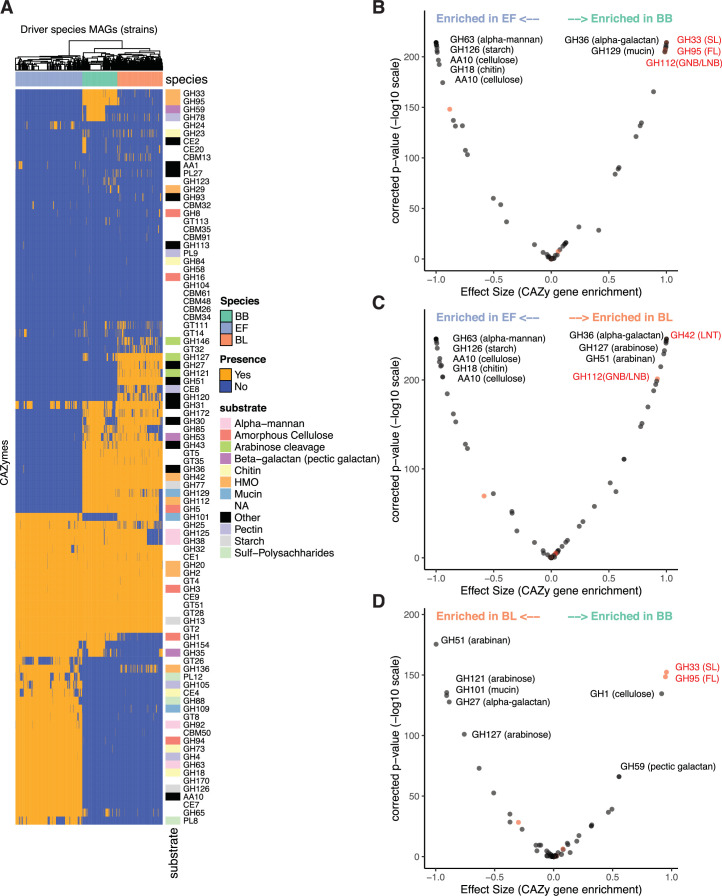
Carbon metabolism of NGM community state driver species. **(a)** A heatmap displays the clustering of carbohydrate-active enzymes (CAZymes) across the genomes of three driver species. Genes are coloured based on their corresponding carbohydrate substrate categories. **(b-d)** Volcano plots depict differentially enriched CAZymes in each driver species, comparing **(b)** BB vs. EF, **(c)** BL vs. EF, and **(d)** BB vs. BL. The effect size represents the difference in the proportion of genes between species. P-values are adjusted using Fisher's exact test (two-sided) with FDR correction. Genes related to HMO metabolism are marked in red. Significantly enriched genes are labelled for clarity. Arrows at the top indicate the direction of species enrichment in each comparison. *B. longum* (BL, N=342) is shown in orange, *B. breve* (BB, N=267) in green, and *E. faecalis* (EF, N=507) in blue.

At the genome-wide functional level, we observed distinct metabolic landscapes of NGM community states based on KEGG orthologues (Extended Data Fig. 8a), particularly in metabolic repertoire of carbohydrate-active enzymes (Extended Data Fig. 8b). Both *Bifidobacterium* community states, in contrast to EF, exhibited an enrichment in carbohydrate-active enzymes associated with metabolizing human milk oligosaccharides (HMOs) abundant and exclusively found in human breast milk. By contrast, EF predominantly possesses genes tailored for utilizing complex dietary glycans such as mannan and chitin, as well as those like starch and cellulose that are commonly found in a plant-based diet usually consumed later in life (Extended Data Figs. 8b and 9).

Compared with the limited HMO metabolic capability of the BL community state, BB is capable of utilizing the all the major HMO substrates including lacto-*N*-tetraose (LNT), lacto-*N*-neotetraose (LNnT) and lacto-*N*-biose (LNB), as well as the primary end-products of HMO metabolism l-fucose and d-lactose, which are naturally present in human breast milk (Fig. 4c). Interestingly, among the three community states, only BB—comprising nearly all *B. breve* genomes (97.6%, *N* = 290/297)—encode the enzyme (α-l-fucosidase, GH95 or GH29) required for metabolizing the most abundant HMO component 2′-fucosyllactose (2′-FL). Although these *B. breve* strains lack known transporters for importing 2′-FL for intracellular metabolism, previous in vitro experiments have shown that similar strains are capable of growing on 2′-FL^32,33^. Therefore, *B. breve* might be able to metabolize 2′-FL via a previously uncharacterized pathway. In contrast, such capability is extremely rare among BL (5.0%, *N* = 19/391) and completely absent in EF (Fig. 4c). Notably, the species-level variations in HMO utilization observed in the study strains are representative of BB/BL/EF species, exhibiting patterns consistent with those previously reported^34^. These patterns are not influenced by breastfeeding rates in this neonatal cohort, which are uniformly high and statistically indistinguishable among the community states (79.1%, 81.8% and 88.6% for EF, BL and BB, respectively).

Given that opportunistic pathogens including *E. faecalis, E. faecium, Klebsiella oxytoca, K. pneumoniae, Enterobacter cloacae* and *Clostridium perfringens*, which are enriched in the EF community state, lack the capability to metabolize HMOs and their by-products, we hypothesize that *B. breve*’s versatility in utilizing these predominant neonatal dietary components substantially enhances its fitness against opportunistic pathogens in vivo. Considering that all neonates in the study would have been exposed to the same level of HMOs through a predominantly breast milk-based diet, regardless of their community state, we reason that the metabolic capability to utilize HMOs, including but not limited to 2′-FL, not only contributes to the dominance and stability of the BB community state but also enables *B. breve* to outcompete pathogenic species that cannot utilize HMOs. Supporting our hypothesis, we demonstrate in a gnotobiotic mouse model, co-colonized with *B. breve* and the opportunistic pathogen driver species *E. faecalis*, that *B. breve* dominates, and this dominance is amplified by dietary 2′-FL supplementation (Fig. 4d). The 2′-FL-mediated pathogen resistance in vivo phenotype of *B. breve* also extends to the Gram-negative enteropathogen *K. oxytoca*, albeit to a lesser extent. Importantly, the anti-pathogen effect was absent in mice colonized with a natural *B. breve* variant isolated from a BBS neonate lacking the α-l-fucosidase (GH95) enzyme necessary for 2′-FL metabolism. These findings suggest that *B. breve*’s strain-specific and gene-dependent utilization of HMOs could have a crucial role in enhancing resistance to pathogen colonization by inhibiting pathogen growth.

## Discussion

In presumably the largest neonatal gut metagenome study ever undertaken, we discovered three distinct NGM community states in over 1,000 healthy, full-term neonates drawn from the general UK population, representing diverse ethnicities and sociodemographic backgrounds. Factors that may influence the maternal gut microbiota, such as maternal age, ethnicity and parity, as well as events that influence its vertical transmission to the neonatal gut during the perinatal period (for example, CS and maternal antibiotics), serve as independent determinants of the acquisition of primary colonizers. The presence of a highly unstable community state (EF) with AMR-enriched opportunistic pathogens underscores the hospital environments and practices, such as maternal antibiotics during labour and elective CS births, as important risk factors^1,35–38^. Although antibiotics after birth and breastfeeding are known important factors shaping the later infant-stage microbiome development^13,15,39,40^, these postnatal factors had no observable effect on very early NGM dynamics on either the acquisition or the switching of the NGM community states. Together, our findings highlight that the NGM assembly outcome is highly dependent on the succession of primary colonizer species, with prenatal and perinatal factors associated with birth exerting profound influences.

Although the early-life microbiota is thought to be highly dynamic as reflected by high inter-individual variation^1^, here we describe an undisturbed, native primary succession pattern in microbiota assembly driven by a single *Bifidobacterium* species. *B. longum* is strongly linked to factors that promote maternal gut microbiota transmission at birth, such as vaginal delivery and absence of antibiotics. While *B. breve* seems unaffected by these factors, its independent association with maternal ethnicity (Asian) could be linked to the mother’s FUT2 secretor status, which determines the presence of 2′-FL and other HMOs in breast milk and is reportedly more common in Asian participants than in white participants^41^. The pattern of exclusive dominance by either *B. breve* or *B. longum* during very early life could also be observed in other cohorts across geographically diverse populations^11–16^. Earlier neonatal cohorts, limited by their smaller sample sizes (*N* < 100 compared with *N* > 1,000 in this study) and lack of longitudinal samplings, were unable to report such patterns as distinctly and conclusively as we have in this study. Given that de novo identification of optimal community state clusters is sample size dependent^10^, our expanded BBS dataset—nearly 10 times larger than the previously largest neonatal dataset^13^—provided us with the statistical power to report a distinct tripartite NGM community structure. This includes a previously undescribed at-risk community state (EF) harbouring AMR-carrying opportunistic pathogens, and presumably for the first time, the epidemiological and longitudinal dynamics signatures of each NGM community state. Our findings provide crucial evidence that can guide the rational selection of species and strains for infant interventional trials, as well as the development of next-generation microbiota-based therapeutics. Future studies can stratify infants by their earliest gut community states to examine potential associations with longer-term health outcomes.

Both *Bifidobacterium* community states can drive deterministic and stable assembly trajectories in vivo through optimized utilization of HMOs exclusively present in human breast milk, the predominant diet during the neonatal period. Our human and in vivo data are in agreement with recent observations based on in vitro experiments^42,43^, showing that *B. breve* is functionally better adapted to an HMO-rich diet in very early life and dominate NGM through priority effects. Here we further demonstrated, in human and mouse, the functional impact of *B. breve* priority effects, resulting in stronger colonization resistance against AMR-enriched pathogens, including *E. faecalis* and *K. oxytoca*.

While the exact origins of opportunistic pathogens such as *E. faecalis* contributing to EF remain to be confirmed, their strong association with disruptions of natural birth (for example, CS and antibiotics) and their ubiquitous presence in the hospital birth environment^22,23^ strongly indicate the hospital operating room as the most likely source, with exposure further exacerbated by the lack of maternal microbiota transmission that frequently occurs during natural birth. Although the EF perturbation patterns appear to be largely transient, with the neonatal microbiota naturally recovering from a delayed colonization trajectory^1,44^, inadequate pathogen clearance could persist into infancy. Along with the short-term exposure to high AMR and virulence, early acquisition of pathogens represents increased risk for infection susceptibility due to the immature immune system in very early life^45^. Also, the delayed or lack of exposure to commensal *B. breve* or/and *B. longum* as a primary colonizer in the critical neonatal window of immunity^45^ and neurological^46^ development could potentially result in neurodevelopment and immune-mediated disorders later in childhood^47^. Epidemiological evidence from other independent birth cohorts indicates that a non-*Bifidobacterium* (for example, EF) community state may predispose neonates to an increased risk of neurological disorders^48^ and respiratory diseases (for example, asthma and atopy^49,50^), including respiratory infections^26,51^, later in childhood.

*Bifidobacterium* spp. are known to achieve bifidogenic effects through the provision of HMOs, with a notable focus on *B. infantis* and its probiotic application as a specialized HMO-utilizing species. Despite its prevalence and dominance in infants from low- to middle-income and non-industrialized settings^17,52^, *B. infantis* is notably absent in this UK cohort and other Western cohorts, suggesting that it may no longer be naturally colonizing newborns in Western, industrialized populations. Its notable absence indicates a potential lack of a reservoir for *B. infantis* to establish itself as a primary colonizer, despite the considerable selective advantage that extensive exposure to HMOs during the neonatal period would presumably provide. Our results demonstrate that an HMO functional niche could be filled by other species (*B. breve* or *B. longum*) capable of metabolizing HMO if they are prevalent in the perinatal microbial species pool. The findings of strain-dependent utilization of HMOs, including but not limited to 2′-FL, and colonization resistance phenotypes of *B. breve* further highlight that the success of primary succession is probably dependent on both the species prevalence and strain-level functional variation.

Maternal seeding of microbial metabolizers of the specialized bioactives in breast milk probably represents an evolutionally conserved strategy to prime human gut microbiota assembly with primary colonizers with the highest likelihood for priority effects, such as *B. breve* and, to a lesser extent, *B. longum*. While both species have been associated with maternal origins^53^, strain transmission analyses from both our work as well as that of others^19^ have identified only *B. longum* as the most frequently transmitted species from the mother’s gut. Although *B. breve* did not appear to originate from the maternal gut microbiota, we cannot rule out the possibility of vertical transmission of very low-abundance *B. breve* strains. Recent cultivation-based evidence has confirmed that such transmission can occur below the limits of metagenomic strain detection^54^. Other unsampled maternal or environmental sources could also be involved in seeding *B. breve*. One likely source is breast milk microbiota, where *B. breve* has been detected and implicated in the entero-mammary pathway—a retrograde mechanism for milk inoculation^21^. Future research should investigate the global strain reservoir and transmission patterns of *Bifidobacterium* species, especially for the poorly understood *B. breve*. Considering the limited success of probiotic-derived *B. infantis* strains in natural engraftment of neonatal gut microbiota in both industrialized and non-industrialized populations^18,55^, comprehensive strain-level functional characterizations of naturally prevalent and stable primary colonizers, such as *B. breve*, are vital. This effort will expedite the discovery of infant probiotics that are better optimized for local populations.

## Methods

### Study population

The Baby Biome Study (BBS) participants were recruited at the Barking, Havering and Redbridge University Hospitals NHS Trust, the University Hospitals Leicester NHS Trust and the University College London Hospitals NHS Foundation Trust from May 2014 to December 2017. The study was approved by the NHS London City and East Research Ethics Committee (REC reference 12/LO/1492). Mothers provided written informed consent for their participation and the participation of their children in the study. The study was performed in compliance with all relevant ethics regulations.

### Whole-genome sequencing and analysis

The study participants, drawn from a general population of women giving birth in hospitals in the UK without any clinical inclusion or exclusion criteria as per the BBS study protocol^56^, are predominantly healthy, full-term neonates. The study dataset comprised 2,387 metagenomes, with 1,679 from the previously published^1^ BBS phase 1 (BBS1) and 708 new neonatal gut metagenomes in BBS phase 2 (BBS2), totalling 1,288 participants. The aim of BBS2 was to sequence all the remaining neonatal samples collected from the original BBS study. The study sample size was predicated on detecting differences by mode of birth rather than providing statistical power to discern differences in microbial community states. The sampling and data processing protocols, ranging from sample collection to sequence data generation, quality control (low-quality trimming and human decontamination) and processing, remained unchanged from those previously described^1^ for BBS1. In brief, faecal samples were collected at home by parents from neonates in the first 3 weeks of life (primarily on days 4, 7 and 21) and later in infancy. Paired maternal faecal samples were taken at the hospital around the time of birth. Most new samples in BBS2 were collected on day 7 of life. The only change was an institute-wide upgrade in the Illumina sequencing platform, transitioning from HiSeq 2500-v4 (2 ×125 bp) to HiSeq 4000 (2 ×151 bp). A multiplexing strategy was employed to ensure that the target depth remained consistent with BBS1. While the upgraded sequencing platform has resulted in a marginal increase in sequencing depth for BBS2 (from 19.3 to 20.4 million reads per sample post-quality control, calculated with seqkit (v.2.4.0)^57^, *P* < 0.001, two-sided *t*-test), it did not impact either the community state assignment (*P* = 0.4731, likelihood ratio test via multinomial logistic regression) or the recovery of high-quality genomes (proportion of the total genome bins) for NGM driver species (*P* = 0.9716, Mantel–Haenszel chi-squared test, stratified by species).

Read-based taxonomic classification was performed against the Genome Taxonomy Database (GTDB, RS207) representative bacterial and archaeal species genomes (*N* = 65,703) using bowtie2 (v.2.3.5)^58^ and inStrain (v.1.3.0)^59^ ‘profile’ with the recommended ‘–database mode’ and 50% genome breadth (covered by ≥1 read) cut-off, as previously described^52,59^. The R package phyloseq (v.1.12.0)^60^ was used for metagenomic data analysis, and results were processed and visualized using tidyverse (v.2.0.0) in RStudio (v.4.1.0).

Strain sharing analysis was performed using StrainPhlAn4 (ref. ^61^), following the workflow and species-specific strain identity thresholds previously described^19^. Where appropriate, multiple testing corrections were applied to all statistical tests using the Benjamini–Hochberg FDR method with a significance threshold of 5%, unless otherwise specified.

Cultivation and whole genome sequencing of the NGM species isolates were performed using the previously established workflow^1^ for BBS1. In brief, the NGM species in driver NGM samples were cultured from corresponding frozen faecal samples using selective media: *Bifidobacterium* selective media (Sigma-Aldrich) for *B. longum* and *B. breve*, and *Enterococcus* selective agar (Sigma-Aldrich) for *E. faecalis*. Purified bacterial isolates were sequenced on the Illumina HiSeq X or NovaSeq 6000 system (2 ×151 bp), and assembled and quality-controlled using shovill (v.1.1.0; https://github.com/tseemann/shovill) and CheckM2 (ref. ^62^), respectively.

### Clinical and sociodemographic metadata sources and management

Participant data were collected using a clinical record form at enrolment by the BBS research midwives or from available clinical records at birth. Hospital maternity electronic records with pregnancy and perinatal clinical information were obtained directly from the hospital trusts, and databases containing the variables of interest were merged. Variables were harmonized where possible across different databases. For discrepancies, data from the BBS clinical record forms were given priority, and hospital electronic data were used to complete missing data. At the time of stool sample collection, mothers completed a short form on feeding mode and antibiotic exposure. A total of 20 variables were included in the final analyses on the basis of clinical relevance, quality of data and completeness (*N* = 6 maternal, *N* = 8 perinatal or at time of delivery, *N* = 5 postnatal, *N* = 1 at the time of stool sample collection variables). Ten variables had no missing or <1% missing data. Four had between <1% and 5% missing data (index of multiple deprivation (IMD), maternal smoking, prolonged rupture of membranes (PROM) and neonatal labour antibiotics after birth), two had between 5% and 15% missing data (maternal ethnicity and feeding mode at the time of stool sample collection), and one had >30% missing data (skin to skin).

We used participant postcode to determine IMD^63^, which provides a measure of socioeconomic status that is calculated as an area-level relative deprivation score that we organized into quintiles from 1 (least deprived) to 5 (most deprived). The score considers seven individually weighted domains (income, employment, education, health, crime, barriers to housing and services, and living environment). Prophylactic antibiotics were administered to all mothers undergoing caesarean section in this cohort, as well as to newborns displaying risk factors or clinical indicators of early-onset neonatal infection, in accordance with local trust policies and UK national guidelines at the time^64,65^. To our knowledge, no participants were given antibiotics for treating bloodstream infections of *E. faecalis*. Skin-to-skin contact is defined as contact of mother and baby immediately after birth at least for 1 h or until the next feeding^66^. Feeding mode at the time of stool sample collection was determined through a questionnaire that included three categories: exclusive breastfeeding, exclusive bottle feeding, or both (that is, mixed feeding). For comparisons involving (non)exclusive breastfeeding, the latter two categories were merged into a single ‘non-exclusive breastfeeding’ category.

### Statistical analyses

No statistical methods were used to pre-determine sample sizes, but this study already represents the largest dataset of longitudinal faecal metagenomes (*n* = 1,904; *n* = 2,387 including infancy samples) of newborns (*n* = 1,288). No data were excluded unless they failed quality control steps. Microbiome data collection and analysis were not randomized or performed blind to the conditions of the experiments, as this is an observational study. Biological counting experiments were blinded by another person other than the experimenter before being counted to avoid experimental bias. For mouse experiments, treatments were randomized by cage by researchers blinded to treatment conditions. Unless otherwise stated, non-parametric statistical tests were performed unless tests for normality and equal variances showed that these assumptions were met.

For the epidemiological analyses of NGM community states in the first week of life, BBS participants with sufficient metadata were explored (90.4%, *N* = 1,108 of 1,225 participants with week-1 sampling). The week-1 NGM community state was determined for each eligible participant by using the earliest available sample from week 1, collected either on day 4 (*N* = 64) or day 7 (*N* = 1,044).

To ascertain risk factors for specific NGM community states: BB versus non-BB, EF versus non-EF, and BL versus non-BL, univariate analyses using fixed-effect logistic regression models were initially performed. Subsequent multivariate models were constructed, also using fixed-effect logistic regression, and included only participants with complete datasets while excluding variables with over 15% missing data. Likelihood ratio tests were employed to calculate all *P* values. A hierarchical framework was applied in building the multivariate models. Variables were organized in a sequential order into either distal (maternal) or more proximal categories (delivery, postnatal care and the first week of life). Variables were considered potential confounders if they occurred simultaneously with or before exposure variables^67^. Within each category, all variables from that category or previous categories were incorporated into the model to account for confounding.

Sensitivity analyses were conducted to identify factors associated with NGM community state, switching between weeks 1 and 3. This included a subset of ‘neonatal longitudinal’ participants with sufficient metadata (87.6%, *N* = 268 of 306, corresponding to Fig. 2a). Both univariate and subsequent multivariate analyses were conducted using fixed-effect logistic regression in the same manner as described above. Multivariate models were further adjusted for the week-1 community state (that is, EF, BB or BL) to discern whether any associations were driven by the baseline community state. There was no strong evidence of association, other than for the baseline community state itself. These analyses could not extend to independent community states switches due to insufficient sample size. All analyses were conducted using Stata (v.17.0).

### Community state assignment

The NGM community state assignment was applied to all neonatal samples (*N* = 1,904) using two popular methods, namely, the original clustering-based PAM method described in ref. ^68^ and the probabilistic modelling-based Dirichlet multinomial mixtures (DMM) approach described previously^69^. In accordance with the original protocols, PAM clustering was applied to the species-level relative abundance distance measured by the Jensen–Shannon divergence (JSD) using the R packages ‘cluster’ (v.2.1.4) and ‘vegan’ (v.2.6.4), and DMM models were fitted on the species-level relative abundance matrix, modelled by the Dirichlet multinomial distribution, using the R package ‘DirichletMultinomial’ (v.1.4). For both methods, the optimal number of clusters of three was determined on the basis of the Calinski–Harabasz index for PAM clustering and the model fit score based on Laplace approximation for DMM. The community states were named according to the top taxonomic driver (species) that contributed the most to microbial community variation (‘envfit’ *R*^2^, *P* < 0.05) in PAM and to each Dirichlet component (cluster) in DMM. The strength of association between the PAM and the DMM-based community states was 0.726 (Cramer’s V correlation). For downstream analyses, the PAM-based community state assignment was selected because it maximized both the sample size of community states BB and BL (Extended Data Fig. 2e) and the mean relative abundance of the driver species in the respective community state (*B. breve* in BB, *E. faecalis* in EF; Extended Data Fig. 2f).

To validate the single-species dominance in external neonatal cohorts, the same workflow for community state type assignment was independently applied to four public gut metagenomic datasets with a comparable sampling window (<6 months) to the BBS cohort, including partial or exclusive sampling of the neonatal period (0–1 month). The earliest sampling windows were from cohorts derived from diverse geographical populations and lifestyles, including Sweden^13,42^ (PRJEB6456, days 4–12, *N* = 37), Israel^14^ (PRJNA994433, weeks 1–24, *N* = 60), the USA (TEDDY cohort^15,16^, PRJNA400115, months 2–6, *N* = 69) and Bangladesh^17^ (PRJNA806984, months 0–2, *N* = 234).

### Metagenome assembly and functional analyses

Quality-controlled, raw paired-end reads were first assembled with SPAdes (v.3.13.5)^70^ with the option –meta. Unassembled reads were then filtered out by mapping raw reads back to metaSPAdes^71^-assembled contigs using bwa-mem (v.0.7.17)^72^, followed by re-assembly with MEGAHIT (v.1.1.3)^73^ using default parameters. Subsequently, the metaSPAdes and MEGAHIT assemblies were combined, sorted and short contigs (<1,500 bp) removed. The resulting assemblies were then independently binned with MetaBAT 2 (v.2.13)^74^, MaxBin2 (v.2.2.4)^75^ and CONCOCT (v.0.4)^76^ using default parameters and a minimum contig length threshold of 1,500 bp (option –minContig 1500). The depth of contig coverage required for the binning was inferred by mapping the raw reads back to their assemblies with bwa-mem (v.0.7.17) and then calculating the corresponding read depths of each individual contig with samtools^77^ (‘samtools view -Sbu’ followed by ‘samtools sort’) together with the ‘jgi_summarize_bam_contig_depths’ function from MetaBAT 2.

Thereafter, individual genome bin sets produced by three binning programs were consolidated into a refined bin set consisting of the best version of each bin based on the most optimal genome completion and contamination metrics among all seven versions of hybridized bin sets (MetaBAT 2, MaxBin2, CONCOCT, MetaBAT 2 + MaxBin2, MetaBAT 2 + CONCOCT, MaxBin2 + CONCOCT, MetaBAT 2 + MaxBin2 + CONCOCT) as estimated by CheckM (v.1.0.7)^78^ using the metaWRAP (v.1.2)^79^ ‘bin_refinement’ pipeline^79^. In total, 22,668 prokaryotic metagenome-assembled genomes (MAGs) met the criteria of having >50% completeness and <5% contamination, as determined by CheckM2 (ref. ^62^). These MAGs were subsequently taxonomically assigned using the GTDB^80^ (R214) taxonomy with GTDB-Tk (v.2.3.0)^81^.

For genome analyses of the three NGM driver species, data were derived from samples as either cultivated isolate genomes or metagenome-assembled genomes (MAGs) when cultured strains were unavailable. Only near-complete, high-quality MAGs were used in the functional analyses (*N* = 1,116). All genomes met strict quality control criteria, which included ≥90% completeness, ≤5% contamination, an N50 value of ≥10 kb, passing the GUNC test, an average contig length of ≥5 kb and ≤500 contigs, as previously described^82^. Genome annotation of metabolic function was performed using DRAM (v.1.4.5)^83^, which integrates annotations from multiple databases, including Pfam, KEGG (KOfam), UniProt, dbCAN (carbohydrate-active enzymes) and MEROPS (peptidases). The functional gene counts from KEGG and CAZy annotations were used to generate a PCA plot using the R package ‘pcaMethods’, employing conventional singular value decomposition with imputation. The genome-based prediction of HMO substrate utilization was based on KEGG and CAZy annotations mapped against a list of manually curated relevant genes and pathways as described recently^42,43^. The genes corresponding to HMO substrates (enzymes; transporters) were: 2′FL (GH95 and/or GH29, FL1_Blon0341-0343 and/or FL2_Blon2202-2204), lactose (GH2, LacS), fucose (FumC/D/E/F/G, FucP), LNT (GH42 or GH136, GltABC), LNnT (GH20, Bbr_1554) and LNB (GH112, GltABC). In silico screening of AMR and virulence factor genes was performed at the species level with species MAGs and at the sample level with raw metagenome assemblies as input for ABRicate against the NCBI AMRFinderPlus and VFDB databases as previously described^1^. The AMR genes encoding for the extended-spectrum β-lactamase (ESBL) phenotype were annotated using the curated antibiotic subclass of the NCBI Pathogen Detection Reference Gene Catalog (as of 1 October 2023).

### Bacterial strains and reagents

The bacterial strains used in this study were either part of the in-house (HMIL) culture collection cultivated from the BBS faecal samples or requested from public collections (DSMZ). Specific strains were: *B. breve* strains (type strain DSM 20213 and D19 isolated from a BBS neonate), *E. faecalis* (D13 isolated from a BBS neonate) and *K. oxytoca* (D63 isolated from a BBS neonate). Purified HMO 2′FL (GlyCare 2FL 9000, batch 20156002) and LNnT were purchased from Glycom, DSM.

### Mouse experiment

Wild-type C57BL/6N mice were maintained under germ-free conditions at the Wellcome Sanger Institute Home Office-approved facility, with all procedures carried out in accordance with the UK Animals (Scientific Procedures) Act of 1986 under Home Office approval (PPL no. 80/2643). Germ-free mice were housed under a 12 h light/12 h dark cycle, ambient temperature and humidity condition in positive-pressure isolators (Bell), with faeces tested by culture, microscopy and PCR to ensure sterility. Consumables were autoclaved at 121 °C for 15 min before introduction into the isolators. For experimentation, 6-week-old mice of both sexes were randomly assigned to treatment groups. Cages were opened in a vaporized hydrogen peroxide-sterilized, class II cabinet (Bioquell), with mono-colonized gnotobiotic lines generated by oral gavage on day 1 (*B. breve*) at the concentration of 10^9^ colony-forming units (c.f.u.) per ml and day 4 (challenged by opportunistic pathogen species *E. faecalis* or *K. oxytoca* at the concentration of 10^4^ c.f.u. per ml). Materials were prepared in Dulbecco’s PBS at 100 mg ml^−1^ immediately before administration under anaerobic conditions (10% H, 10% CO_2_, 80% N) in a Whitley DG250 workstation at 37 °C. Mice were maintained in sterile ISOcages (Tecniplast) and housed on ISOrack for the period of the experiment.

Control groups of mice colonized with BB, EF or KO without any treatment were also included to confirm mono-colonization. One of the two groups of the co-colonized mice (for both BB + EF and BB + KO experiments) were exposed to 2′-FL via daily drinking water (50 mg ml^−1^ per day) throughout the experiment. Faecal samples were collected on each oral gavage day and plated to test for contamination. Mice were killed on day 11 (7 days post inoculation on day 4), with faecal samples collected and plated for colony count on yeast extract casitone fatty acids (YCFA) aerobically (to select for *E. faecalis* or *K. oxytoca*) and YCFA with mupirocin (to select for *Bifidobacterium* spp.) media under anaerobic conditions. YCFA is a complex, broad-range medium^84^. Each experimental condition included 3–5 mice per cage and 3 technical replicate cages.

DNA was extracted from faeces using FastDNA Spin Kit for Soil (MPBio) according to manufacturer instructions, and DNA eluted into 100 µl of double-distilled H_2_O. Eluted DNA was then diluted 1:50 and qPCR performed using SYBR Green chemistry (Thermo Fisher). The absolute bacterial load in each faecal sample was determined by qPCR using a calibration curve generated with genomic DNA and taxon-specific primer sequences (*E. faecalis*, F: 5′-CCCTTATTGTTAGTTGCCATCATT-3′, R: 5′-ACTCGTTGTACTTCCCATTGT-3′; *Bifidobacterium* spp., F: 5′-CTCCTGGAAACGGGTGG-3′, R: 5′-GGTGTTCTTCCCGATATCTACA-3′; *K. oxytoca*, F: 5′-GGACTACGCCGTCTATCGTCAAG-3′, R: 5′- TAGCCTTTATCAAGCGGATACTGG-3′). As previously described^85^, the relative abundance of each target species was estimated by normalizing to those of a universal bacterial 16S primer (F: 5′-GTGSTGCAYGGYTGTCGTCA-3′, R: 5′-ACGTCRTCCMCACCTTCCTC-3′).

### Reporting summary

Further information on research design is available in the Nature Portfolio Reporting Summary linked to this article.

## Supplementary information

Reporting Summary

Supplementary Data Table of contents tab. Supplementary Table 1. Sample_accession. ENA accessions of the 2,387 samples of entire BBS cohort analysed in this manuscript: BBS1 (N = 1,679) and BBS2 (N = 708). 2. Neonatal_subject. Clinical metadata of the BBS neonates included in the statistical analysis (N = 1,108), cohort characteristics described in Extended Data Table 1. 3. Neonatal_sample. Sample metadata (age/day and community states) of the neonatal samples included in the analyses. N = 1,904. 4. Epi_metadata_summary. Descriptive table of the BBS neonatal population with available metadata (N = 1,108/1,288, 90%). 5. Epi_result_neonatal_state. Clinical and sociodemographic variables associated with the acquisition of NGM community state measured in the first week of life (N = 1,108). 6. Epi_result_neonatal_switch. Clinical and sociodemographic variables associated with NGM community state switching between week 1 and week 3 (N = 306). 7. Genome_accession. Sample accessons of the species genomes generated from the BBS samples and analysed in the functional analyses.

## Data Availability

Shotgun metagenomic sequencing data (after quality trimming and human decontamination) of the entire Baby Biome Study cohort have been deposited to the European Nucleotide Archive under study accession number ERP115334. Bacterial genome assemblies for the three species analysed have been deposited in Zenodo at 10.5281/zenodo.12667210 (ref. ^86^). Sample metadata and participant-level clinical metadata of de-identified study participants are provided in the Supplementary Tables. The raw faecal samples and bacterial isolates are available from the corresponding authors upon request.

## Code availability

All software used to perform these analyses is publicly available. Software tools used are listed in the main text and Methods.
