# Supplementary material for: Primary succession of Bifidobacteria drives pathogen resistance in neonatal microbiota assembly
Source: Nat Microbiol. 2024 Sep 6;9(10):2570–82. doi: 10.1038/s41564-024-01804-9 (PMC11445081; doi:10.1038/s41564-024-01804-9)
Supplement: Supplementary file 1 — Reporting Summary [file 41564_2024_1804_MOESM1_ESM.pdf]

nature portfolio

Corresponding author(s): Yan Shao
Trevor D. Lawley

Last updated by author(s): Jul 5, 2024

Reporting Summary

Nature Portfolio wishes to improve the reproducibility of the work that we publish. This form provides structure for consistency and transparency in reporting. For further information on Nature Portfolio policies, see our Editorial Policies and the Editorial Policy Checklist.

Statistics

For all statistical analyses, confirm that the following items are present in the figure legend, table legend, main text, or Methods section.

n/a	Confirmed	
☐	☒	The exact sample size (*n*) for each experimental group/condition, given as a discrete number and unit of measurement
☐	☒	A statement on whether measurements were taken from distinct samples or whether the same sample was measured repeatedly
☐	☒	The statistical test(s) used AND whether they are one- or two-sided<br>*Only common tests should be described solely by name; describe more complex techniques in the Methods section.*
☐	☒	A description of all covariates tested
☐	☒	A description of any assumptions or corrections, such as tests of normality and adjustment for multiple comparisons
☐	☒	A full description of the statistical parameters including central tendency (e.g. means) or other basic estimates (e.g. regression coefficient) AND variation (e.g. standard deviation) or associated estimates of uncertainty (e.g. confidence intervals)
☐	☒	For null hypothesis testing, the test statistic (e.g. *F*, *t*, *r*) with confidence intervals, effect sizes, degrees of freedom and *P* value noted<br>*Give P values as exact values whenever suitable.*
☒	☐	For Bayesian analysis, information on the choice of priors and Markov chain Monte Carlo settings
☒	☐	For hierarchical and complex designs, identification of the appropriate level for tests and full reporting of outcomes
☒	☐	Estimates of effect sizes (e.g. Cohen's *d*, Pearson's *r*), indicating how they were calculated

Our web collection on statistics for biologists contains articles on many of the points above.

Software and code

Policy information about availability of computer code

Data collection	No software was used for data collection.
Data analysis	All software used for data analyses is publicly available, and listed as follows: InStrain v1.3.0, bowtie2 v2.3.5, StrainPhlAn4 v4.0.6, phyloseq v1.48, RStudio v4.1.0, R packages cluster v2.1.4, DirichletMultinomial v1.4, tidyverse v2.0.0, ggalluvial v0.12.5, lmerTest v3.1-3, Prism 9, seqkit v2.4.0, shovill v1.1.0, SPAdes v3.15.5, MEGAHIT v1.1.3, bwa-mem v0.7.17, MetaBAT2 v2.13, MaxBin2 v2.2.4 and CONCOCT v0.4, samtools v1.5, metaWRAP v1.2, CheckM2 v1.0.1, GTDB-Tk v2.3.0., DRAM v1.4.5, ABRicate v1.0.1

For manuscripts utilizing custom algorithms or software that are central to the research but not yet described in published literature, software must be made available to editors and reviewers. We strongly encourage code deposition in a community repository (e.g. GitHub). See the Nature Portfolio guidelines for submitting code & software for further information.

Data

Policy information about availability of data

All manuscripts must include a data availability statement. This statement should provide the following information, where applicable:

- Accession codes, unique identifiers, or web links for publicly available datasets
- A description of any restrictions on data availability
- For clinical datasets or third party data, please ensure that the statement adheres to our policy

Shotgun metagenomic sequencing data (after quality trimming and human decontamination) of the entire Baby Biome Study cohort have been deposited to the

European Nucleotide Archive under study accession number ERP115334. Bacterial genome assemblies for the three species analysed have been deposited to Zenodo (https://doi.org/10.5281/zenodo.12667210). Sample metadata and subject-level clinical metadata of de-identified study participants are provided in the Supplementary Tables. The raw faecal samples and bacterial isolates are available from the corresponding authors upon request.

Research involving human participants, their data, or biological material

Policy information about studies with human participants or human data. See also policy information about sex, gender (identity/presentation), and sexual orientation and race, ethnicity and racism.

Reporting on sex and gender	Sex of the study participants was collected in this study based on the clinical records at birth. Sex has been included as a variable in the molecular epidemiological analyses, with cohort-level data summarized in Table 1, and individual-level data listed in Supplementary Table 1.
Reporting on race, ethnicity, or other socially relevant groupings	Maternal ethnicity of the study participants was collected in this study based on a self-reported questionnaire at recruitment. Maternal ethnicity has been included as a variable in the molecular epidemiological analyses, with cohort-level data summarized in Table 1, and individual-level data listed in Supplementary Table 1.
Population characteristics	All clinical covariates of the study cohort are summarised in Table 1. A total of 20 variables were included in the final analyses based on clinical relevance, quality of data and completeness (N=6 maternal, N=8 perinatal or at time of delivery, N=5 postnatal, N=1 at the time of stool sample collection variables).
Recruitment	Participants were recruited on a voluntary basis in the study hospitals. Mothers provided written informed consent for their participation, and for the participation of their children, in the study.
Ethics oversight	The study was approved by the NHS London – City and East Research Ethics Committee (REC reference 12/LO/1492). The study was performed in compliance with all relevant ethical regulations.

Note that full information on the approval of the study protocol must also be provided in the manuscript.

Field-specific reporting

Please select the one below that is the best fit for your research. If you are not sure, read the appropriate sections before making your selection.

☒ Life sciences ☐ Behavioural & social sciences ☐ Ecological, evolutionary & environmental sciences

For a reference copy of the document with all sections, see nature.com/documents/nr-reporting-summary-flat.pdf

Life sciences study design

All studies must disclose on these points even when the disclosure is negative.

Sample size	All fecal samples collected in this study were used if available. No statistical methods were used to pre-determine sample sizes, but this study already represents the largest dataset of longitudinal fecal metagenomes (n = 1,904; n = 2,387 including infancy samples) of newborn babies (n = 1,288).
Data exclusions	None. All sequencing samples that passed sequencing quality control were included for analysis.
Replication	All experimental data presented include replicates, with the number of biological replicates stated in the figure captions.
Randomization	Randomization was not employed in microbiome analyses as this is an observational study. Mice were allocated randomly into three replicate cages per experimental condition.
Blinding	No blinding used in microbiome analyses as this is an observational study. Biological counting experiments were blinded by another person than the experimenter before being counted as to avoid experimental bias.

Reporting for specific materials, systems and methods

We require information from authors about some types of materials, experimental systems and methods used in many studies. Here, indicate whether each material, system or method listed is relevant to your study. If you are not sure if a list item applies to your research, read the appropriate section before selecting a response.

Materials & experimental systems

n/a	Involved in the study
☒	☐ Antibodies
☒	☐ Eukaryotic cell lines
☒	☐ Palaeontology and archaeology
☐	☒ Animals and other organisms
☒	☐ Clinical data
☒	☐ Dual use research of concern
☒	☐ Plants

Methods

n/a	Involved in the study
☒	☐ ChIP-seq
☒	☐ Flow cytometry
☒	☐ MRI-based neuroimaging

Animals and other research organisms

Policy information about studies involving animals; ARRIVE guidelines recommended for reporting animal research, and Sex and Gender in Research

Laboratory animals	Wild-type C57BL/6N mice; median age 6 weeks; SD 12 days.
Wild animals	This study did not involve wild animals.
Reporting on sex	No sex-based analyses have been performed. Cages of male and female mice were randomly allocated to experimental groups.
Field-collected samples	This study did not involve field-collected samples.
Ethics oversight	Mice were maintained under germ-free conditions at the Wellcome Sanger Institute Home Office-approved facility, with all procedures carried out in accordance with the United Kingdom Animals (Scientific Procedures) Act of 1986 under Home Office approval (PPL no. 80/2643).

Note that full information on the approval of the study protocol must also be provided in the manuscript.

Plants

Seed stocks	*Report on the source of all seed stocks or other plant material used. If applicable, state the seed stock centre and catalogue number. If plant specimens were collected from the field, describe the collection location, date and sampling procedures.*
Novel plant genotypes	*Describe the methods by which all novel plant genotypes were produced. This includes those generated by transgenic approaches, gene editing, chemical/radiation-based mutagenesis and hybridization. For transgenic lines, describe the transformation method, the number of independent lines analyzed and the generation upon which experiments were performed. For gene-edited lines, describe the editor used, the endogenous sequence targeted for editing, the targeting guide RNA sequence (if applicable) and how the editor was applied.*
Authentication	*Describe any authentication procedures for each seed stock used or novel genotype generated. Describe any experiments used to assess the effect of a mutation and, where applicable, how potential secondary effects (e.g. second site T-DNA insertions, mosiacism, off-target gene editing) were examined.*
